# Efficacy of a novel mode of action of an indoor residual spraying product, SumiShield® 50WG against susceptible and resistant populations of *Anopheles gambiae* (*s.l.*) in Benin, West Africa

**DOI:** 10.1186/s13071-018-2869-6

**Published:** 2018-05-10

**Authors:** Fiacre R. Agossa, Gil G. Padonou, Come Z. Koukpo, Jacques Zola-Sahossi, Roseric Azondekon, Osei K. Akuoko, Juniace Ahoga, Boris N’dombidje, Bruno Akinro, Arsene Jacques Y. H. Fassinou, Michel Sezonlin, Martin C. Akogbeto

**Affiliations:** 1 Centre de Recherche Entomologique de Cotonou (CREC), Cotonou, Bénin; 2 Laboratoire Evolution, Biodiversité des Arthropodes et Assainissement, FAST – UAC, Abomey-Calavi, Bénin; 3 Ecole Doctorale Sciences de la Vie et de la Terre, FAST – UAC, Abomey-Calavi, Bénin; 4 Noguchi Memorial Institute for Medical Research University, Accra, Ghana

**Keywords:** *Anopheles gambiae* (*s.l.*), SumiShield 50WG, Clothianidin, Experimental hut, Efficacy, Covè, Benin

## Abstract

**Background:**

Scale-up of the distribution of long-lasting insecticide-treated bed nets and indoor residual spraying with insecticides over the last decade have contributed to the considerable decrease of malaria morbidity and mortality in sub-Saharan Africa. Due to the increasing pyrethroid resistance intensity and the spread of carbamate resistance in *Anopheles gambiae* (*s.s.*) mosquitoes and the limited number of insecticides recommended by the WHO for vector control, alternative insecticide formulations for IRS with long-lasting residual activity are required to sustain the gains obtained in most malaria-endemic countries.

**Methods:**

SumiShield 50WG (clothianidin 300 mg ai/m^2^) developed by Sumitomo Chemical was evaluated alongside deltamethrin 25 mg ai/m^2^ (K-Othrine 250 WG) against a pyrethroid resistant *Anopheles gambiae* (*s.l.*) population in experimental huts in Covè, Benin. Residual activity was also tested in cone bioassays with the susceptible *An. gambiae* “Kisumu” strain and the local wild resistant population.

**Results:**

The results showed very low toxicity from deltamethrin (mortality rates ranged between 1–40%) against host-seeking resistant *Anopheles* populations. SumiShield in contrast gave an overall mean mortality of 91.7% at the 120 h observation across the eight- month observation period following spraying. The residual activity measured using cone tests was over the 80% WHO threshold for 24 weeks for resistant wild *Anopheles* population and 32 weeks for the susceptible strain “Kisumu” after the spraying.

**Conclusions:**

SumiShield is a good candidate for IRS in areas of permanent malaria transmission and where *Anopheles* populations are resistant to other conventional insecticides such as pyrethroids. It would be interesting to complete experimental huts studies by assessing the efficacy and residual effect of SumiShield 50WG at community level (small-scale field testing) in an area where vectors are highly resistant to insecticides.

## Background

The first lesson learnt from the monitoring and evaluation of indoor residual spraying (IRS) in Africa is the variation in the residual life of the main insecticides used for indoor residual spraying: bendiocarb (carbamate) and pirimiphos methyl (organophosphate). Actellic EC (pirimiphos methyl) and Ficam VC (bendiocarb) have shown a short residual effect in several countries such as Benin, Mali, Rwanda and Equatorial Guinea [1–4]. In previous IRS programs, pirimiphos methyl capsule suspension (CS) showed a longer residual effect in Ghana compared to other countries such as Benin, Ethiopia, Liberia, Mali, Senegal and Zimbabwe [4]. The second problem is the emergence of carbamate and organophosphate resistance [5]. Pyrethroid resistance is widespread in most malaria endemic countries and since carbamate resistance has arisen in some countries, only organophosphates, the remaining class of insecticides recommended by the WHO, is used by National Malaria Control Programmes for indoor residual spraying and for resistance management. For the National Malaria Control Programmes and the international community, it is judicious to prevent the rapid and widespread resistance to carbamates and organophosphates by evaluating new insecticide formulations with different modes of action and a long residual effect.

The solution proposed by the WHO for resistance management [6] appears difficult to implement due to the limited number of insecticides recommended for IRS. The mitigation of the spread of vector resistance is to avoid subjecting *Anopheles* to the same products or those with the same mode of action over several years or to reduce resistance selection pressures in order to weaken the resistance gene carriers toward these products. In the Atacora, Alibori and Donga areas in Benin where pirimiphos methyl CS is the insecticide used for IRS, the NMCP recommends its usage for not more than two or three years and suggests the implementation strategy for resistance management based on the rotational use of two or three insecticides with different modes of action.

Overall, given the limited number of insecticides recommended for LLINs and IRS since the 1970s and malaria vectors developing resistance to most insecticide classes, formulations of insecticides with new modes of action are needed for resistance management. The Innovative Vector Control Consortium (IVCC) has developed novel active ingredients. These new candidates should be on the market by or around 2022 for IRS. Also, new formulations and new classes of insecticides could be recommended for LLINs impregnation.

SumiShield® 50WG (clothianidin) developed by Sumitomo, is a good candidate as needed by all NMCPs in Africa, and has prequalification listing. SumiShield® contains clothianidin, a neonicotinoid insecticide not previously used for vector control. As a result, it is expected that pyrethroid resistant mosquito populations should be killed by SumiShield 50WG. This study was implemented to evaluate the efficacy and residual effect of SumiShield 50WG applied at 300 mg ai/m^2^ against wild pyrethroids resistant *Anopheles* population in semi-field conditions in experimental huts in Benin, West Africa.

## Methods

### Study design

The study was conducted in the experimental station at Covè, Southern Benin, over a period of 8 months (October 2015 to May 2016). The huts were built alongside a paddy field which constitutes the permanent breeding sites for *Anopheles* mosquitoes. Adult volunteers were recruited among the inhabitants of the location (Covè). Pregnant and breast-feeding women were not involved in the study. After having announced through the district for volunteers, selection was done after approval was granted by the traditional head of the study site.

Sleepers (mosquito collectors) were rotated randomly among huts each night following a Latin square design. They entered the huts at dusk (21:00 h) and remained inside until dawn. In the morning at 6:30 h, dead mosquitoes were collected from the floor of the huts and resting mosquitoes from the walls, roof and exit traps using aspirators. Mosquitoes were scored by location as dead or alive and as fed or unfed. Live mosquitoes were placed in small cups and provided with 10% sugar solution for 24, 48, 72, 96 and 120 h to assess delayed mortality. All wild *An. gambiae* (*s.l.*) mosquitoes caught per night and per hut were taken into account. In each month, sleepers collected free-flying host-seeking *Anopheles gambiae* (*s.l.*) from Covè during four Latin square weeks per month (24 days). One Latin square week was equal to six days. A sleeper slept once in each of the six huts used during six successive nights and rested on the seventh night. The wild mosquito populations were complemented with the susceptible *An. gambiae* “Kisumu” strain which were reared in the insectary of the Centre de Recherche Entomologique de Cotonou (CREC) and released in the huts. At least 20 females of *An. gambiae* “Kisumu” strain aged 5–8 days were released in each hut over one night per month; these numbers were kept reasonably low to prevent sleepers from receiving too many mosquito bites.

The primary parameters measured in these experimental huts according to the duration of the trial were: (i) deterrence: reduction in hut entry relative to the control huts (untreated hut); (ii) induced exophily: the proportion of mosquitoes that exited early and were found in exit traps; (iii) blood-feeding inhibition: the reduction in blood-feeding compared with that in the control hut; (iv) immediate mortality: the proportion of mosquitoes that were killed at the end of the exposure time; and (v) delayed mortality: the proportion of mosquitoes that were killed after 24, 48, 72, 96 and 120 h.

**Fig. 1:**
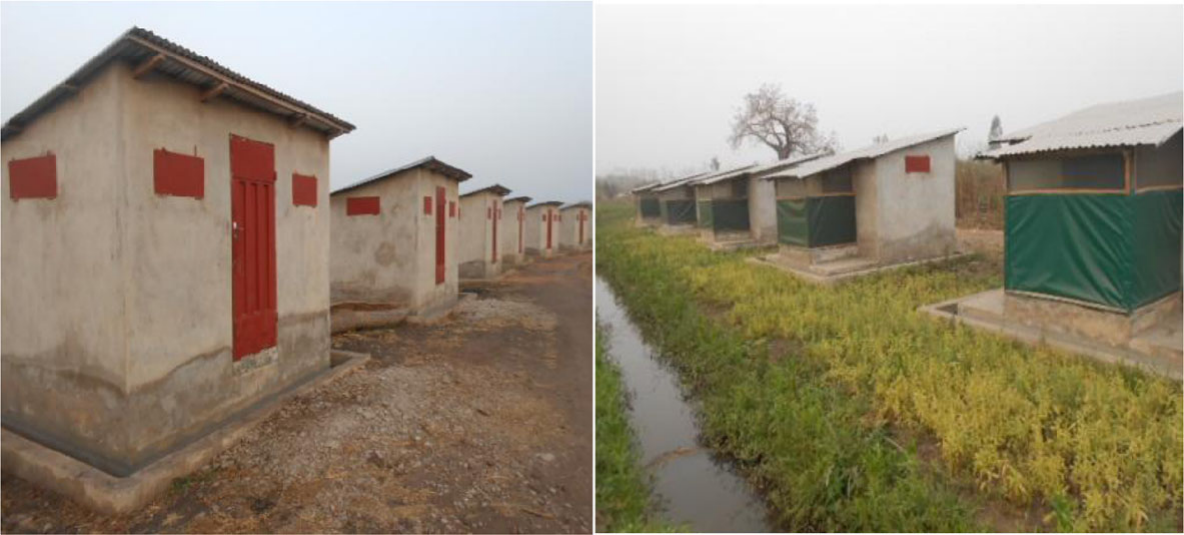
Experimental hut station at Covè, Benin

The huts used were designed to simulate local and typical West African households (Fig. 1). The description and how the host-seeking mosquitoes were captured as described by [7]. Six experimental huts (smooth cement) were used in this study: 2 huts for SumiShield 50WG (contains 50% w/w clothianidin), 2 huts for K-Othrine 250WG (Deltamethrin 250 WG, contains 25% w/w deltamethrin) and 2 untreated (control).

### Insecticide treatment

The maximum safety instructions and protective measures were observed. The operators who treated the walls wore full protective clothing: long-sleeved shirt and trousers, hat, rubber boots, gloves and a particle-filtering half-mask.

#### Dilution process

Standard nozzles recommended by the WHO for malaria vector control are types 8002 or 8001. A standard nozzle type 8002 was used. This nozzle delivers a volume of 760 ml of insecticide solution / min (for 19 m^2^). If a sprayer does not have a CFV (control flow valve), an average application rate of 40 ml/m^2^ is assumed. In the present study, the nozzle was accompanied by a red CFV (1.5 bar pressure) which delivers a volume of 570 ml of insecticide solution/min (for 19 m^2^) or 30 ml/m^2^.

(i) Dilution of SumiShield 50WG. The capacity of the sprayer used (Hudson Xpert, Chicago, USA) was 6 l. The sprayer was rinsed with clean water after each treatment. For a volume of 6 l, the dilution was: Q = (300 mg × 6000 ml) / 30 ml = 60,000 mg of clothianidin in 6 l of water. The SumiShield 50WG contained 50% clothianidin. Therefore 120 g (120,000 mg) of SumiShield 50WG was diluted in 6 l of water to treat 200 m^2^. To manage the remaining diluted insecticide after treatment, each insecticide solution was adjusted to each hut surface area. For hut 1 (17 m^2^), the dilution was 10,200 mg SumiShield 50WG in 510.0 ml of water. For hut 5 (16.74m^2^), dilution was 10,044 mg SumiShield 50WG in 502.0 ml of water

(ii) Dilution of K-Othrine 250WG. For a volume of 6 l of the sprayer (Hudson Xpert) used, the dilution was: Q = (6000 ml × 25 mg) / 30 ml = 5000 mg of deltamethrin in 6 l of water. K-Othrine 250WG contained 25% of deltamethrin; therefore, the dilution was 20 g (20,000 mg) of K-250 WG Othrine in 6 l of water to treat 200 m^2^. To manage the rest of diluted insecticide after treatment, each insecticide solution was adjusted to each hut surface area.For hut 3 (16.86 m^2^), the dilution was 1686 mg of K-250 WG Othrine in 506 ml of water. For hut 4 (16.00 m^2^), the dilution was 1600 mg of K-250 WG Othrine in 480 ml of water. Huts 2 and 6 were used as control (untreated).

### Measurement of pH of the wall substrates

The purpose of this activity was to intermittently follow the evolution of the pH of the wall surfaces and the efficacy of the impregnated insecticides as well. A small quantity (5 g of substrate) from the wall surface of each hut was scraped into a Petri dish before, during and at the end of the study. The substrates were dissolved in distilled water for the determination of the pH in CREC.

### Status of resistance of the wild *An. gambiae* population of the study area

Susceptibility tests using WHO tubes were performed using 2–5 day-old aged adult female mosquitoes. Detection of Leu-Phe *kdr* mutation was performed with untested mosquito samples using PCR following the protocol described by [8].

### WHO cone bioassay

For each month, WHO cone bioassays were performed. A laboratory colony of *An. gambiae* “Kisumu” strain which were fully susceptible to all insecticides and a wild population of *An. gambiae* (*s.l.*) resistant to pyrethroids sampled around the study site were used. The WHO cone bioassays [9] were carried out in week 1, and each month after spraying. During the whole study, cones were placed at fixed level (0.5, 1, 1.5 and 2 m) on the walls (treated and untreated huts). Each level was randomly selected and labelled to one wall surface of each hut. For each month, at least 10 females of the *An. gambiae* susceptible reference strain “Kisumu” and wild *An. gambiae* (*s.l.*) mosquitoes aged 3–5 days were introduced per cone for 30 min exposure. After this exposure period, knockdown mosquitoes were recorded and those alive were kept in observation for 24, 48, 72, 96 and 120 h to score delayed mortalities. Mosquitoes exposed to unsprayed substrates were used as controls. When the mortality in the control was between 5 and 20%, corrected mortality was determined using Abbot’s formula.

### Statistical analysis

The raw data were managed with Microsoft Excel and the statistical analyses were performed using R software. The WHO criteria were used to classify the level of resistance of tested mosquitoes from Covè to insecticides. The WHO bio-efficacy threshold was used for the analyses of the residual effect in time per treatment. Others analyses on the significance between each of the bio-efficacy measured parameters in treated huts were made and compared between treatment and control (untreated) using R software.

## Results

### Analysis of pH of samples of wall substrates

Analysis of the pH of wall samples of the huts was done 7 days prior to spraying, and 4 and 8 months after spraying in the huts. The mean pH observed is indicated in Table 1. No significant variation of mean pH was observed between insecticide treatments at the observation times (4 and 8 months after spraying).

**Table 1:** pH measured on walls prior to spraying, 4 and 8 months after spraying

Treatment	Structure ID	Time	Respective pH values	Mean pH	SD
Control	H2 and H6	T0	7 ; 7	7	0
Control	H2 and H6	T4	7 ; 7	7	0
Control	H2 and H6	T8	7 ; 7	7	0
Deltamethrin	H3 and H4	T0	7 ; 6	6.5	0.70
Deltamethrin	H3 and H4	T4	7 ; 8	7.5	0.70
Deltamethrin	H3 and H4	T8	9 ; 8	8.5	0.70
SumiShield	H1 and H5	T0	6 ; 6	6	0
SumiShield	H1 and H5	T4	8 ; 8	8	0
SumiShield	H1 and H5	T8	7 ; 10	8.5	2.12

*Abbreviations*: *H2* and *H6* controls 1 and 2, *H3* and *H4* deltamethrin 1 and 2, *H1* and *H5* SumiShield 1 and 2, *T0* prior to spraying, *T4* 4 months after spraying, *T8* 8 months after spraying, *SD* standard deviation

### Resistance level of *An. gambiae* (*s.l.*) population from the experimental huts station

**Fig. 2:**
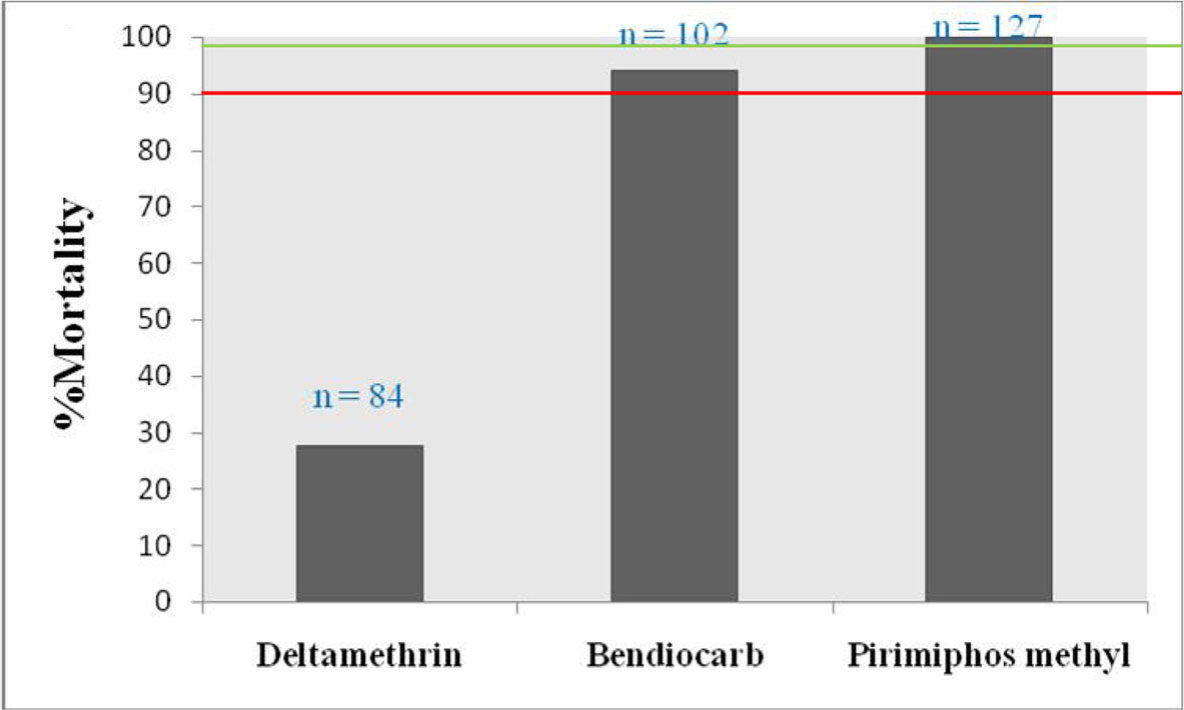
Status of resistance of *Anopheles* population to deltamethrin, bendiocarb and pirimiphos methyl in the experiment huts station of Covè, Benin, October 2015

Prior to spraying, the resistance status of the population of *An. gambiae* (*s.l.*) from the study site was investigated. The results showed that the *Anopheles* population from the experimental hut station was resistant to deltamethrin 0.05% (mortality rate: 27.45%) and susceptible to pirimiphos methyl 0.25% (mortality rate: 100%). A resistance to bendiocarb 0.1% was also suspected (Fig. 2).

### Resistance mechanisms in *Anopheles* population

PCR results showed a high frequency (0.95) of knockdown resistance (*kdr*) and very low frequency (0.03) of acetylcholinesterase insensitive mutation (*Ace-1*) in the *Anopheles* population from the experimental hut station (Table 2).

**Table 2:** Target site mutation frequencies (*kdr and Ace-1*) in the experimental hut station in Covè, Benin

No. tested	*kdr* mutation				*Ace-1* mutation			
	RR	RS	SS	F (*kdr*)	RR	RS	SS	F (*Ace-1*)
30	27	3	0	0.95	0	2	28	0.03

*Abbreviations*: *RR* homozygote resistant, *RS*heterozygote resistant, *SS* homozygote susceptible, *F* (*kdr*) frequency of *kdr* mutations, *F* (*Ace-1*) frequency of *Ace-1* mutations

### Efficacy of clothianidin 50WG against host-seeking *Anopheles* populations in the experimental huts station

Over the period of eight months of evaluation of the efficacy of clothianidin 50WG in comparison to that of deltamethrin 250WG, 24,135 host-seeking *An. gambiae* (*s.l.*) were collected from the six experimental huts used in this study. Among these mosquitoes, 35.7% (8609), 31.4% (7577) and 33.0% (7949) were collected from the untreated huts (control huts), the huts treated with clothianidin and those treated with deltamethrin, respectively. The results of the measured parameters are shown below.

#### Exophily

Induced exophily is the proportion of mosquitoes that exited the huts and were thus found in the exit trap veranda. Overall, all treatments induced in the host-seeking *An. gambiae* (*s.l.*) population an exit behavior from the treated surfaces to the exit trap’s veranda (Table 3). However, significantly higher exophily rates were observed with deltamethrin (*P* < 0.0001).

**Table 3:** Exophily rates observed per month and per treatment with wild host-seeking *Anopheles* populations

Treatment	Month	Total number	Proportion	95% CI	*P-*value^a^
Control 1	1	386	33.68	28.96–38.39	–
	2	563	28.60	24.86–32.33	–
	3	630	20.16	17.03–23.29	–
	4	379	30.34	25.71–34.97	–
	5	411	33.82	29.25–38.39	–
	6	445	29.89	25.63–34.14	–
	7	647	32.77	29.15–36.38	–
	8	802	23.19	20.27–26.11	–
Control 2	1	531	30.51	26.59–34.42	0.31
	2	731	29.96	26.64–33.28	0.59
	3	722	25.62	22.44–28.81	0.02
	4	523	30.78	26.83–34.74	0.89
	5	481	27.86	23.85–31.87	0.05
	6	427	28.57	24.29–32.86	0.67
	7	475	34.32	30.05–38.59	0.59
	8	456	36.84	32.41–41.27	< 0.0001
SumiShield 1	1	379	33.51	28.76–38.26	0.96
	2	453	33.33	28.99–37.67	0.10
	3	683	20.35	17.33–23.37	0.93
	4	426	26.53	22.33–30.72	0.23
	5	365	26.58	22.04–31.11	0.03
	6	424	28.54	24.24–32.84	0.66
	7	484	34.71	30.47–38.95	0.49
	8	614	36.64	32.83–40.46	< 0.0001
SumiShield 2	1	317	33.12	27.94–38.30	0.88
	2	513	29.24	25.30–33.18	0.82
	3	500	31.40	27.33–35.47	< 0.0001
	4	370	32.70	27.92–37.48	0.49
	5	439	34.62	30.17–39.07	0.80
	6	431	31.79	27.39–36.18	0.54
	7	562	37.01	33.02–41.08	0.12
	8	617	40.36	36.49–44.23	< 0.0001
Deltamethrin 1	1	450	77.56	73.70–81.41	< 0.0001
	2	523	75.33	71.64–79.03	< 0.0001
	3	454	61.67	57.20–66.15	< 0.0001
	4	482	70.75	66.69–74.81	< 0.0001
	5	405	73.09	68.77–77.41	< 0.0001
	6	469	65.25	60.94–69.55	< 0.0001
	7	560	57.50	53.41–61.59	< 0.0001
	8	652	55.21	51.40–59.03	< 0.0001
Deltamethrin 2	1	388	79.12	75.08–83.17	< 0.0001
	2	542	75.09	71.45–78.73	< 0.0001
	3	487	62.42	58.12–66.72	< 0.0001
	4	369	78.05	73.83–82.27	< 0.0001
	5	441	74.15	70.06–78.24	< 0.0001
	6	490	64.90	60.67–69.12	< 0.0001
	7	637	61.54	57.76–65.32	< 0.0001
	8	600	58.67	54.73–62.61	< 0.0001

*Abbreviation*: *CI* confidence interval

^a^5% significance threshold

#### Blood-feeding

Blood-feeding inhibition describes the reduction in blood-feeding compared with that in the control huts. Irrespective of whether the huts were treated or not, no difference was observed on the blood-feeding of mosquitoes in the experimental huts (Table 4). The blood-feeding rates were over 80% in the treated and control huts.

**Table 4:** Blood-feeding observed with wild host-seeking *Anopheles* populations

Treatment	Month	Total number	Proportion	95% CI	*P*-value^a^
Control 1	1	386	91.97	89.26–94.68	–
	2	563	94.32	92.40–96.23	–
	3	630	93.02	91.03–95.01	–
	4	379	88.92	85.76–92.08	–
	5	411	93.19	90.75–95.62	–
	6	445	97.98	96.67–99.29	–
	7	647	89.64	87.30–91.99	–
	8	802	96.63	95.39–97.88	–
Control 2	1	531	93.97	91.95–95.70	0.24
	2	731	92.48	90.56–94.39	0.19
	3	722	84.63	81.99–87.26	< 0.0001
	4	523	92.73	90.51–94.96	0.05
	5	481	96.67	95.07–98.28	0.02
	6	427	92.27	89.74–94.80	< 0.0001
	7	475	94.95	92.98–96.92	< 0.0001
	8	456	94.08	91.91–96.25	0.03
SumiShield 1	1	379	94.46	92.16–96.76	0.17
	2	453	92.27	89.81–94.73	0.19
	3	683	91.36	89.25–93.47	0.27
	4	426	96.01	94.15–97.87	< 0.0001
	5	365	96.99	95.23–98.74	0.02
	6	424	96.70	94.53–98.02	0.24
	7	484	94.63	92.62–96.64	< 0.0001
	8	614	84.36	81.49–87.24	< 0.0001
SumiShield 2	1	317	90.85	87.68–94.03	0.60
	2	513	90.45	87.90–92.99	0.02
	3	500	89.80	87.15–92.45	0.05
	4	370	92.62	88.80–94.44	0.21
	5	439	97.95	96.62–99.28	< 0.0001
	6	431	95.36	93.37–97.35	0.03
	7	562	95.37	93.64–97.11	< 0.0001
	8	617	88.01	85.44–90.57	< 0.0001
Deltamethrin 1	1	450	88.00	84.67–90.69	0.06
	2	523	93.12	90.95–95.29	0.41
	3	454	91.41	88.83–93.99	0.33
	4	482	95.64	93.82–97.47	< 0.0001
	5	405	98.02	96.67–99.38	< 0.0001
	6	469	96.16	94.42–97.90	0.11
	7	560	89.82	87.32–92.33	0.92
	8	652	91.87	89.77–93.97	< 0.0001
Deltamethrin 2	1	388	88.66	85.50–91.81	0.12
	2	542	90.77	88.34–93.21	0.02
	3	487	89.94	87.27–92.61	0.06
	4	369	95.39	93.25–97.53	< 0.0001
	5	441	98.19	96.94–99.43	< 0.0001
	6	490	97.96	96.71–99.21	0.98
	7	637	92.62	90.59–94.65	0.06
	8	600	93.83	91.91–95.76	0.01

*Abbreviation*: *CI* confidence interval

^a^5% significance threshold

#### Toxicity effect

**Fig. 3:**
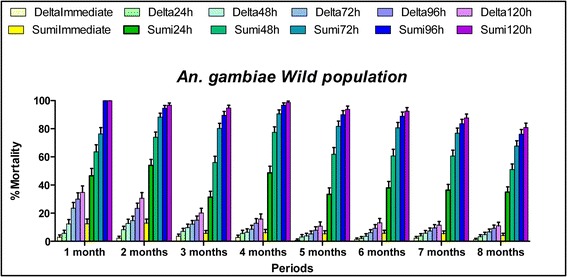
Mortality rates recorded per observation time and per treatment against free-flying, host-seeking *Anopheles* population from Covè. SumiImmediate and Sumi24h, Sumi48h, Sumi72h, Sumi96h and Sumi120h refer to the mortality rates observed each month 24, 48, 72, 96 and 120 h observation times in the huts treated with SumiShield, respectively. DeltaImmediate and Delta24h, Delta48h, Delta72h, Delta96h and Delta120h refer to the mortality rates observed each month 24, 48, 72, 96 and 120 h observation times in the huts treated with deltamethrin, respectively

The toxicity or lethal property of deltamethrin was low (ranging between 1 and 40%). The mortality rates recorded with this insecticide decreased after one month (Fig. 3). However, high mortality rates (mean mortality rate of 91.7% at the 120 h observation point) were recorded over a period of eight months on the evaluation with clothianidin. While good initial mortality was observed, mortality continued to increase across observation times to 120 h indicating the additional killing properties of clothianidin.

### Efficacy of clothianidin 50WG against laboratory susceptible *Anopheles* populations in the experimental huts station

**Fig. 4:**
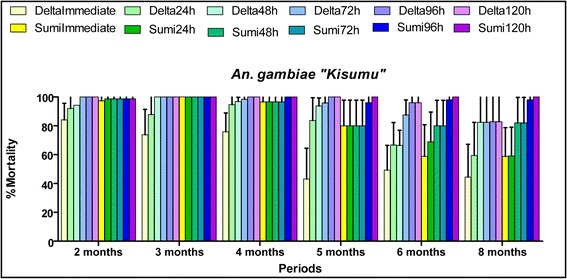
Mortality rates recorded per observation time and per treatment against laboratory susceptible *Anopheles* “Kisumu” strain released per month in the huts. SumiImmediate and Sumi24h, Sumi48h, Sumi72h, Sumi96h and Sumi120h refer to the mortality rates observed each month 24, 48, 72, 96 and 120 h observation times in the huts treated with SumiShield, respectively. DeltaImmediate and Delta24h, Delta48h, Delta72h, Delta96h and Delta120h refer to the mortality rates observed each month 24, 48, 72, 96 and 120 h observation times in the huts treated with deltamethrin, respectively

The results from releasing activities are summarized in Tables 5, 6 and Fig. 4. The exophily and blood-feeding rates observed in the huts treated either with clothianidin or deltamethrin were not significantly higher than those observed in the untreated huts (Tables 5, 6). However, clothianidin induced high mortality rates (about 100%) post-120 h observation time over a period of seven months of the evaluation. The induced mortality rate by deltamethrin decreased to about 80% at month 8. The additional killing effect of clothianidin over time against the susceptible *Anopheles* population released in the huts was also noticed (Fig. 4).

**Table 5:** Exophily rates observed per treatment from month 2 to 8 after treatment with laboratory susceptible *Anopheles* populations released in the huts

Treatment	Month	Total number	Proportion	95% CI	*P*-value^a^
Control 1	2	30	66.67	42.81–90.52	–
	3	27	62.96	44.75–81.18	–
	4	26	26.92	9.87–43.97	–
	5	13	53.85	26.75–80.95	–
	6	32	28.13	12.55–43.70	–
	8	31	38.71	21.56–55.86	–
Control 2	2	32	68.75	46.04–91.46	0.90
	3	44	50.00	35.23–64.77	0.29
	4	30	50.00	32.11–67.89	0.08
	5	25	28.00	10.40–45.60	0.12
	6	49	36.73	23.24–50.23	0.42
	8	26	57.69	38.70–76.68	0.15
SumiShield 1	2	56	75.00	63.66–86.34	0.52
	3	25	44.00	24.54–63.46	0.17
	4	35	45.71	29.21–62.22	0.13
	5	17	70.59	48.93–92.25	0.35
	6	27	37.04	18.82–55.25	0.47
	8	14	64.29	39.19–89.39	0.11
SumiShield 2	2	27	48.15	29.30–67.00	0.25
	3	12	8.33	0.30–23.97	< 0.0001
	4	49	16.33	5.98–26.68	0.27
	5	24	45.83	25.90–65.77	0.64
	6	38	63.16	47.82–78.50	< 0.0001
	8	22	72.73	54.12–91.34	0.01
Deltamethrin 1	2	47	48.94	34.64–63.23	0.23
	3	40	55.00	39.58–70.42	0.52
	4	32	46.88	29.58–64.17	0.12
	5	17	58.82	35.43–82.22	0.79
	6	13	46.15	19.05–73.25	0.24
	8	25	44.00	24.54–63.46	0.69
Deltamethrin 2	2	21	61.90	41.13–82.68	0.77
	3	18	33.33	11.56–55.11	0.05
	4	42	0		< 0.0001
	5	19	47.37	24.92–69.82	0.72
	6	32	59.38	42.36–76.39	0.01
	8	22	68.18	48.72–87.65	0.03

*Abbreviation*: *CI* confidence interval

^a^5% significance threshold

**Table 6:** Blood-feeding rate observed per treatment from month 2 to 8 after treatment with laboratory susceptible *Anopheles* populations released in the huts

Treatment	Month	Total number	Proportion	95% CI	*P*-value^a^
Control 1	2	30	100	92.3–100	–
	3	27	51.85	33.99–69.26	–
	4	26	65.38	47.10–83.67	–
	5	13	69.23	44.14–94.32	–
	6	32	96.88	0.85–98.33	–
	8	31	90.32	75.10–96.65	–
Control 2	2	32	75.00	53.78–96.22	0.04
	3	44	88.64	79.26–98.01	< 0.0001
	4	30	63.33	46.09–80.58	0.87
	5	25	92.00	75.03–97.78	0.07
	6	49	97.96	89.31–99.64	0.76
	8	26	57.69	38.70–76.68	< 0.0001
SumiShield 1	2	56	80.36	69.95–90.76	0.06
	3	25	64.00	45.18–82.82	0.38
	4	35	48.57	32.01–65.13	0.19
	5	17	82.35	64.23–99.20	0.4
	6	27	88.89	77.03–96.30	0.22
	8	14	42.86	16.93–68.78	< 0.0001
SumiShield 2	2	27	51.85	33.99–69.26	< 0.0001
	3	12	33.33	6.66–60.01	0.28
	4	49	30.61	17.71–43.52	< 0.0001
	5	24	70.83	52.65–89.02	0.92
	6	38	68.42	53.64–83.20	< 0.0001
	8	22	90.91	72.19–97.47	0.94
Deltamethrin 1	2	47	19.15	7.90–30.40	< 0.0001
	3	40	57.50	42.18–72.82	0.65
	4	32	53.13	35.83–70.42	0.35
	5	17	64.71	41.99–87.42	0.79
	6	13	38.46	12.01–64.91	< 0.0001
	8	25	48.00	28.42–67.58	< 0.0001
Deltamethrin 2	2	21	9.52	3.03–22.08	< 0.0001
	3	18	16.67	0.55–33.88	0.02
	4	42	30.95	16.97–44.93	0.01
	5	19	15.79	0.61–32.19	< 0.0001
	6	32	100	97.12–100	0.31
	8	22	9.09	1.53–21.10	< 0.0001

*Abbreviation: CI* confidence interval

^a^5% significance threshold

### Residual effect of clothianidin 50WG against susceptible strain “Kisumu” and wild in experimental huts

#### Cone bioassay results

**Fig. 5:**
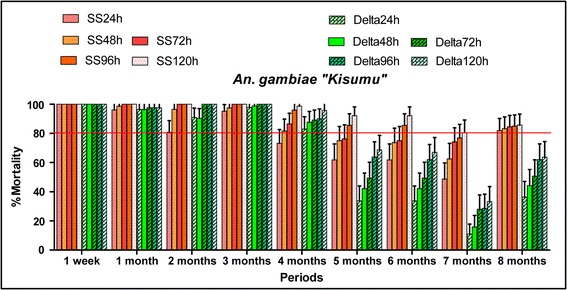
Residual effect of SumiShield 50WG and deltamethrin 250 WG represented by the mortality rates observed following cone bioassays against laboratory susceptible strain “Kisumu” in the experimental hut of Covè. Sumi24h, Sumi48h, Sumi72h, Sumi96h and Sumi120h refer to the mortality rates observed each month 24, 48, 72, 96 and 120 h observation times in the huts treated with SumiShield, respectively. Delta24h, Delta48h, Delta72h, Delta96h and Delta120h refer to the mortality rates observed each month 24, 48, 72, 96 and 120 h observation times in the huts treated with deltamethrin, respectively

**Fig. 6:**
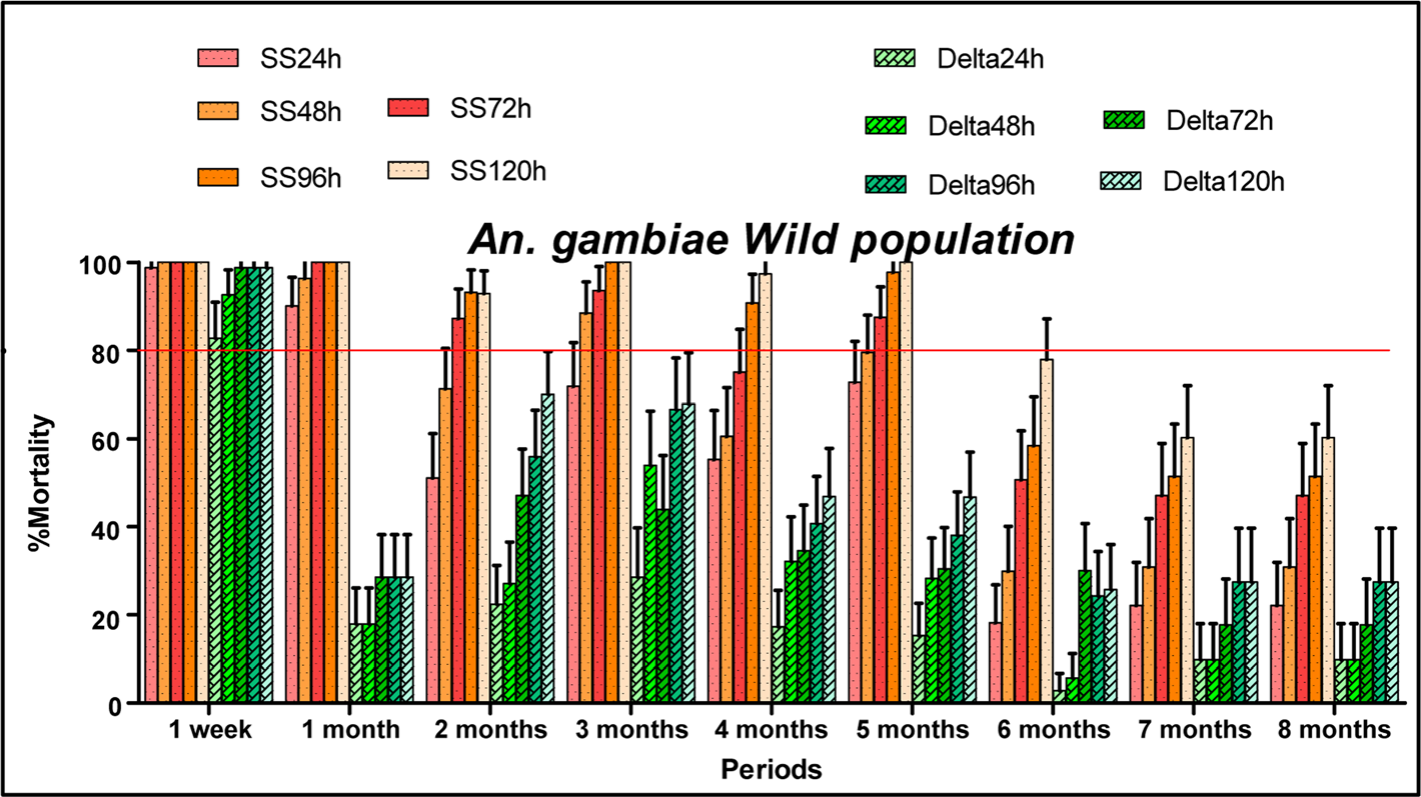
Residual effect of SumiShield 50WG and deltamethrin 250 WG represented by the mortality rates observed following cone bioassays against wild resistant *Anopheles gambiae* (*s.s.*)in the experimental hut of Covè. Sumi24h, Sumi48h, Sumi72h, Sumi96h and Sumi120h refer to the mortality rates observed each month 24, 48, 72, 96 and 120 h observation times in the huts treated with SumiShield, respectively. Delta24h, Delta48h, Delta72h, Delta96h and Delta120h refer to the mortality rates observed each month 24, 48, 72, 96 and 120 h observation times in the huts treated with deltamethrin, respectively

The efficacy of each treatment in terms of mortality rate observed after exposure of mosquitoes to treated walls was compared to the WHO bio-efficacy threshold [9], 80% and to that recorded in the untreated huts. The mortality rates over 5% in control huts were corrected using Abbott’s formula. During the eight months of the evaluation, the huts treated with clothianidin showed the mortality rates over 80% at the 120 h observation point against susceptible *Anopheles* population “Kisumu” (Fig. 5). Concerning the wild *An. gambiae* (*s.l.*) population from Covè (experimental hut station) the mortality rates over 80% were observed until six months in the huts treated with clothianidin (Fig. 6. The mortality rate decreased a little below 80% from the seventh month (77.9%) to the eighth month (60.3%). Mortality rates increased each month across observation times demonstrating the additional time-dependent killing effect of clothianidin.

#### Knockdown effect

**Fig. 7:**
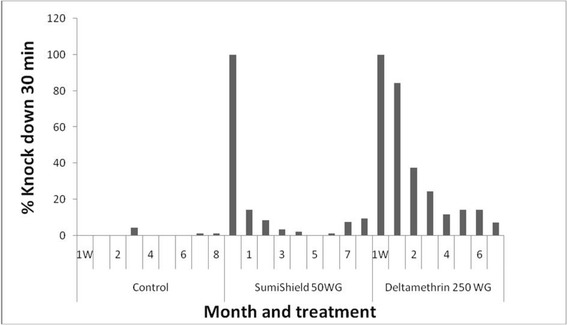
Efficacy represented by knockdown rate at 30 min after WHO cone bioassay per month, per observation time with SumiShield 50WG and deltamethrin 250 WG against susceptible strain “Kisumu” *Anopheles gambiae* (*s.s.*) 1M, 2M, 3M, 4M, 5M, 6M and 7M refer to 1, 2, 3, 4, 5, 6 and 7 months, respectively.

The efficacy of each treatment in terms of knockdown rate obtained after 30 min exposure in cone bioassays of mosquitoes to treated walls was compared to the WHO threshold bio-efficacy [9] which is 95% for LLINs, and to that observed in the untreated huts. Overall, the knockdown rates in the structures treated with clothianidin were very low (< 95% for most) compared with those obtained in the huts treated with deltamethrin (Fig. 7).

## Discussion

The present study assessed the efficacy and residual effect of a novel mode of action Indoor Residual Spray product, SumiShield 50WG under semi-field conditions in West Africa (Covè, Bénin) where *An. gambiae* (*s.l.*) is resistant to pyrethroids. SumiShield was evaluated against a population of *An. gambiae* characterized by a high frequency (0.95) of knockdown resistance (*kdr*) and elevated oxidase and glutathione S-transferase (*GST*) activities. After eight months of evaluation, SumiShield 50WG showed a better efficacy and lasting residual effect compared to deltamethrin. The results were encouraging since deltamethrin was ineffective against a pyrethroid resistant host-seeking *Anopheles* population from Covè during the whole study period.

The exophily of host-seeking wild *An. gambiae* (*s.l.*) recorded with deltamethrin was significantly higher than what was observed in the control huts. This is not unexpected for pyrethroids. During the evaluation period, the blood-feeding rates were high in both treated and control huts. Irrespective of the huts treated or not, the blood-feeding behavior was the same. This is, however, not worrying; many phase II and phase III evaluations implemented in Benin continuously demonstrated that despite the treatment of the houses with insecticides, the majority of mosquitoes successfully enter these treated houses and take their blood meal of their host before resting on the treated walls [10, 11]. Indeed, this is not unexpected for IRS treatments except for DDT that has some spatial action as well as contact activity. This finding explains why it was proposed to Benin NMCP to always educate communities who are protected by IRS to additionally sleep under LLINs to supplement and maximize malaria control efforts. Such combination strategy implicates an increase in the cost of malaria prevention, but it is desirable for areas with high levels of malaria transmission. Fortunately, 95% of blood-fed *An. gambiae* (*s.l.*) died after resting on the treated walls in huts treated with clothianidin 50WG. This will keep them from staying alive to continue malaria transmission. Furthermore, community-wide use of SumiShield 50WG in IRS will produce a “mass effect” on the reduction of the density of infective mosquitoes in the area and, consequently, protecting the whole community including those whose houses are not treated.

Given that SumiShield 50WG has demonstrated at least eight months (study period) efficacy in this study, this insecticide could be a good solution for IRS in areas of permanent malaria transmission. Clothianidin is a novel neonicotinoid insecticide acting as an agonist of the nicotinic acetylcholine receptor (nAChR). This receptor is different from those of the existing recommended insecticide families (organochlorine, pyrethroids, carbamates and organophosphates). So far, good performance of clothianidin against a resistant *Anopheles* population was demonstrated in cement built experimental huts of Malanville, Benin (Corbel, 2012, personal communication). The residual effect observed is superior to that of insecticides recommended by the WHO for indoor residual spraying [8]. The knockdown rates observed with clothianidin were low: this is not unexpected given the mode of action of this insecticide. Additionally, clothianidin has been intensively used in agriculture. Several studies have demonstrated that clothianidin is highly active not only against hemipteran insects but also coleopteran, thysanopteran, dipteran and some lepidopteran pests [12]. Because of its broad spectrum of insecticidal activity, good systemic properties and low mammalian toxicity, clothianidin is a compound that is considered to be compatible with integrated pest management strategies [13].

The use of the new cost-effective, long-lasting IRS insecticides with a new mode of action such as SumiShield for malaria control or elimination in endemic countries will help support resistance management and the optimization of vectors control strategies. However, NMCPs should recommend the judicious use of these new insecticides in preventing the early selection and development of resistance in these malaria parasite vectors.

The additional killing effect of clothianidin over time against both susceptible and resistant *Anopheles* populations was noticed. Given this, it is important to study the impact of this action on the fertility of female malaria vectors subjected to clothianidin in terms of the number of eggs laid and the viability of the embryo from the egg. Furthermore, as the performance of clothianidin as observed in this study was beyond six months, it would also be important to implement another study at community level (small scale Phase III trial) to assess the efficacy and residual effect of SumiShield 50WG under field conditions.

## Conclusions

After eight months evaluation in semi-field conditions, a good efficacy and residual effect of SumiShield 50WG against both susceptible and pyrethroid resistant *Anopheles* population were observed. This insecticide with a novel mode of action for vector control could be a good alternative for IRS in areas of permanent malaria transmission and where mosquitoes are resistant to other insecticides.

## Abbreviations

CFV: Control flow valve
CREC: Centre de Recherche Entomologique de Cotonou
CS: Capsule suspension
DDT: Dichlorodiphenyltrichloroethane
EC: Emulsifiable concentrate
IRS: Indoor residual spraying
IVCC: Innovative Vector Control Consortium
KDR: Knockdown resistance
LLIN: Long-lasting insecticidal net
NMCP: National Malaria Control Programme
WG: Water dispersible granules
WHO: World Health Organization
